# Supplementary material for: Estimating the differences in critical thermal maximum and metabolic rate of Helicoverpa punctigera (Wallengren) (Lepidoptera: Noctuidae) across life stages
Source: PeerJ. 2021 Nov 17;9:e12479. doi: 10.7717/peerj.12479 (PMC8605760; doi:10.7717/peerj.12479)
Supplement: Supplemental Information 3 [file peerj-09-12479-s003.pdf]

Table 1: Percentage weight (mg) loss in three different life stages of *H. punctigera* over the complete ramping period of about 120mins from 25°C to CT_{max} following thermolimit respirometry.

Life Stage	Weight loss (% Mean± SE)
Larvae	22.5±1.0a
Pupae	1.9±0.9b
Adult	17.9±1.2c

Means within columns are significantly different (p>0.05, Tukey-Kramer LSD mean separation).
Note: n=10 in each life stage
