# Supplementary material for: Estimating the differences in critical thermal maximum and metabolic rate of Helicoverpa punctigera (Wallengren) (Lepidoptera: Noctuidae) across life stages
Source: PeerJ. 2021 Nov 17;9:e12479. doi: 10.7717/peerj.12479 (PMC8605760; doi:10.7717/peerj.12479)
Supplement: Supplemental Information 4 [file peerj-09-12479-s004.pdf]

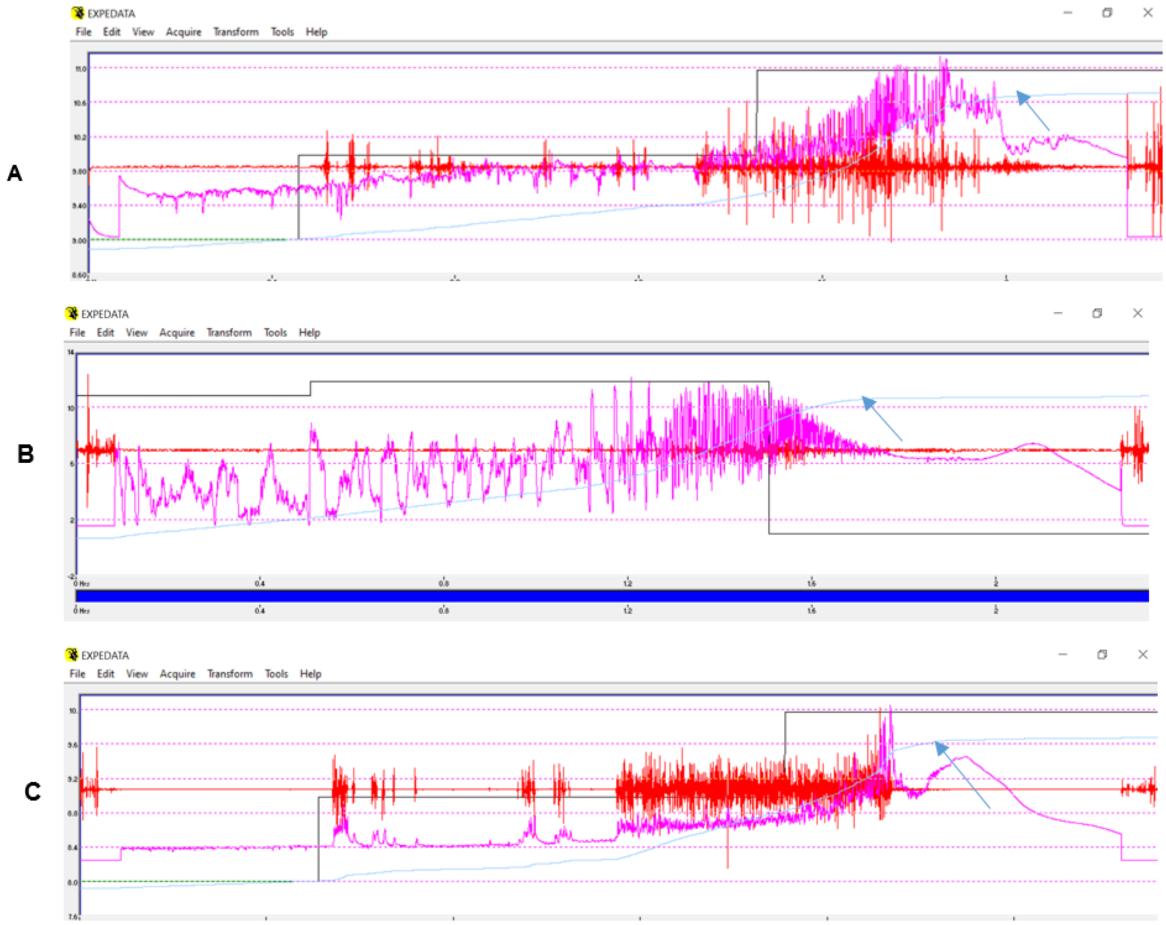

Figure 1: Expedata representative trace from a thermolimit respirometry trial of *H. punctigera* life stages (A: Larva; B: Pupa and C: Adult). VCO2 (pink) and activity (Red) are shown over the complete ramping period of about 140mins from 25°C to 55°C. The blue arrow indicates CT_{max} measured at the cessation of spiracular activity
