# Supplementary material for: Estimating the differences in critical thermal maximum and metabolic rate of Helicoverpa punctigera (Wallengren) (Lepidoptera: Noctuidae) across life stages
Source: PeerJ. 2021 Nov 17;9:e12479. doi: 10.7717/peerj.12479 (PMC8605760; doi:10.7717/peerj.12479)
Supplement: Supplemental Information 5 — Effect of different temperature phases on VCO2in H. punctigera life stages (Larvae, Pupae and Adult) during thermolimit respirometry. Points are means ± s.e.m. [file peerj-09-12479-s005.pdf]

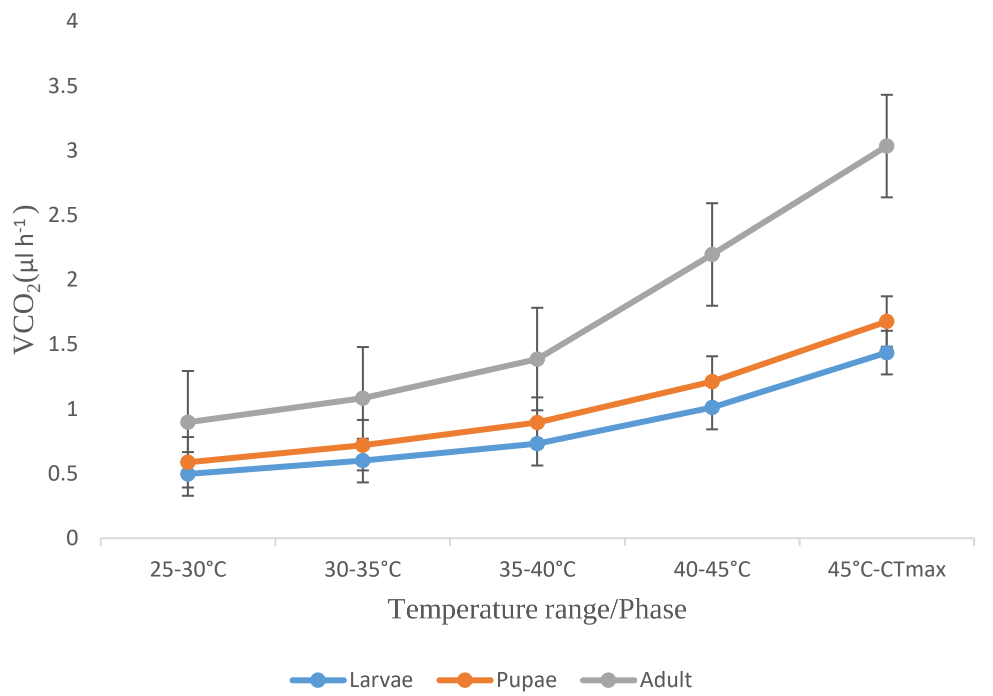

Figure 2. Effect of different temperature phases on VCO_2 in *H. punctigera* life stages (Larvae, Pupae and Adult) during thermolimit respirometry. Points are means ± s.e.m.
