# Supplementary material for: Estimating the differences in critical thermal maximum and metabolic rate of Helicoverpa punctigera (Wallengren) (Lepidoptera: Noctuidae) across life stages
Source: PeerJ. 2021 Nov 17;9:e12479. doi: 10.7717/peerj.12479 (PMC8605760; doi:10.7717/peerj.12479)
Supplement: Supplemental Information 6 — Box plots with different letters are significantly different (p > 0.05). [file peerj-09-12479-s006.pdf]

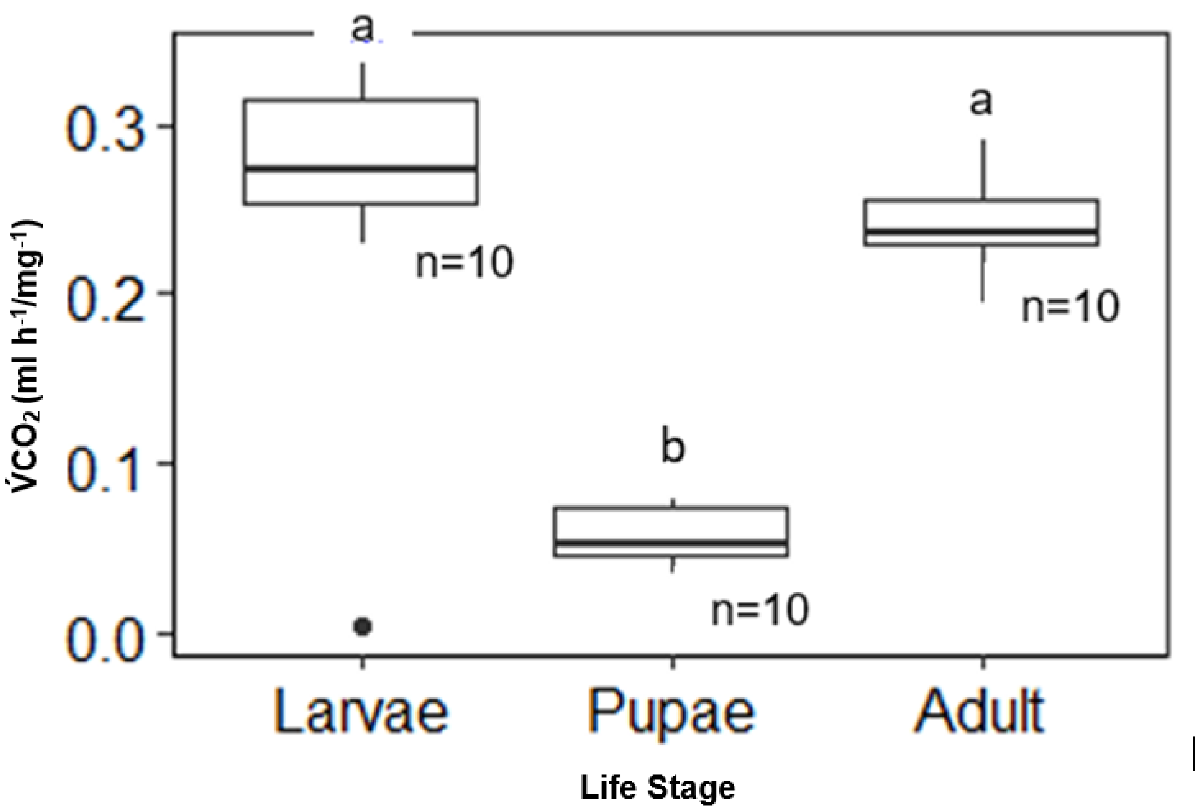

Figure 3: Mass-specific metabolic rate $\acute{V}CO_2$ (ml h^{-1}/mg^{-1}) of *H. punctigera* life stages (larvae, pupae and adult) over the complete ramping period of about 120mins from 25°C to CT_{max} following thermolimit respirometry. Box plots are significantly different ($p>0.05$).
