# Supplementary material for: Estimating the differences in critical thermal maximum and metabolic rate of Helicoverpa punctigera (Wallengren) (Lepidoptera: Noctuidae) across life stages
Source: PeerJ. 2021 Nov 17;9:e12479. doi: 10.7717/peerj.12479 (PMC8605760; doi:10.7717/peerj.12479)
Supplement: Supplemental Information 8 [file peerj-09-12479-s008.docx]

anova <- aov(Z$CTmax~Z$Life_stage+Z$Before_wt_mg)

Summary (anova)

|  | Df | Sum Sq | Mean Sq | F value | Pr(>F) |
| --- | --- | --- | --- | --- | --- |
| Life stage | 2 | 16.785 | 8.392 | 23.536 | 4.34e-06 *** |
| Before weight | 1 | 0.131 | 0.131 | 0.367 | 0.551 |
| Residuals | 21 | 7.488 | 0.357 |  |  |

anova <- aov(Z$CTmax~Z$Life_stage*before_weight)

summary (anova)

|  | Df | Sum Sq | Mean Sq | F value | Pr(>F) |
| --- | --- | --- | --- | --- | --- |
| Life stage | 2 | 16.785 | 8.392 | 24.719 | 5.16e-06*** |
| Before weight | 1 | 0.131 | 0.131 | 0.385 | 0.542 |
| Life Stage*before weight | 2 | 1.037 | 0.519 | 1.528 | 0.243 |
| Residuals | 19 | 6.451 | 0.340 |  |  |
